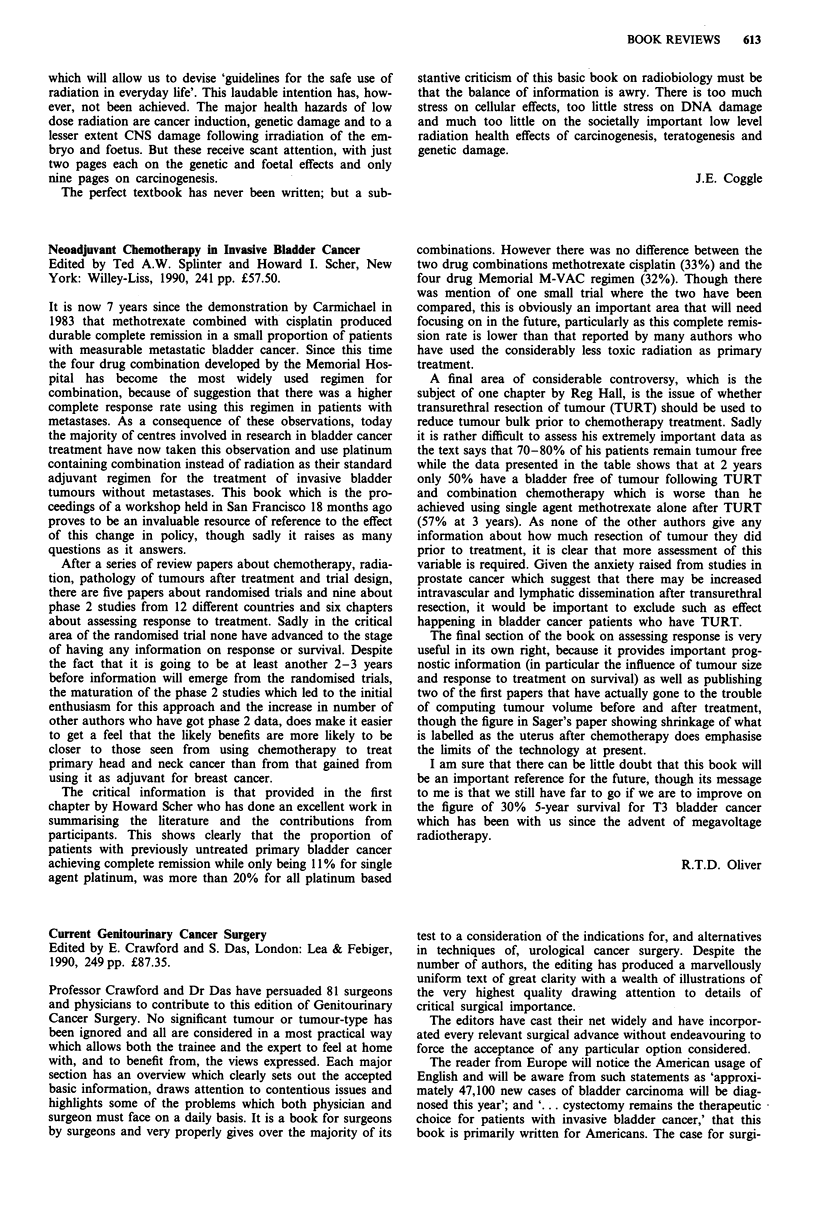# Neoadjuvant Chemotherapy in Invasive Bladder Cancer

**Published:** 1991-09

**Authors:** R.T.D. Oliver


**Neoadjuvant Chemotherapy in Invasive Bladder Cancer**
Edited by Ted A.W. Splinter and Howard I. Scher, New York: Willey-Liss, 1990, 241 pp. £57.50.

It is now 7 years since the demonstration by Carmichael in 1983 that methotrexate combined with cisplatin produced durable complete remission in a small proportion of patients with measurable metastatic bladder cancer. Since this time the four drug combination developed by the Memorial Hospital has become the most widely used regimen for combination, because of suggestion that there was a higher complete response rate using this regimen in patients with metastases. As a consequence of these observations, today the majority of centres involved in research in bladder cancer treatment have now taken this observation and use platinum containing combination instead of radiation as their standard adjuvant regimen for the treatment of invasive bladder tumours without metastases. This book which is the proceedings of a workshop held in San Francisco 18 months ago proves to be an invaluable resource of reference to the effect of this change in policy, though sadly it raises as many questions as it answers.

After a series of review papers about chemotherapy, radiation, pathology of tumours after treatment and trial design, there are five papers about randomised trials and nine about phase 2 studies from 12 different countries and six chapters about assessing response to treatment. Sadly in the critical area of the randomised trial none have advanced to the stage of having any information on response or survival. Despite the fact that it is going to be at least another 2–3 years before information will emerge from the randomised trials, the maturation of the phase 2 studies which led to the initial enthusiasm for this approach and the increase in number of other authors who have got phase 2 data, does make it easier to get a feel that the likely benefits are more likely to be closer to those seen from using chemotherapy to treat primary head and neck cancer than from that gained from using it as adjuvant for breast cancer.

The critical information is that provided in the first chapter by Howard Scher who has done an excellent work in summarising the literature and the contributions from participants. This shows clearly that the proportion of patients with previously untreated primary bladder cancer achieving complete remission while only being 11% for single agent platinum, was more than 20% for all platinum based combinations. However there was no difference between the two drug combinations methotrexate cisplatin (33%) and the four drug Memorial M-VAC regimen (32%). Though there was mention of one small trial where the two have been compared, this is obviously an important area that will need focusing on in the future, particularly as this complete remission rate is lower than that reported by many authors who have used the considerably less toxic radiation as primary treatment.

A final area of considerable controversy, which is the subject of one chapter by Reg Hall, is the issue of whether transurethral resection of tumour (TURT) should be used to reduce tumour bulk prior to chemotherapy treatment. Sadly it is rather difficult to assess his extremely important data as the text says that 70–80% of his patients remain tumour free while the data presented in the table shows that at 2 years only 50% have a bladder free of tumour following TURT and combination chemotherapy which is worse than he achieved using single agent methotrexate alone after TURT (57% at 3 years). As none of the other authors give any information about how much resection of tumour they did prior to treatment, it is clear that more assessment of this variable is required. Given the anxiety raised from studies in prostate cancer which suggest that there may be increased intravascular and lymphatic dissemination after transurethral resection, it would be important to exclude such as effect happening in bladder cancer patients who have TURT.

The final section of the book on assessing response is very useful in its own right, because it provides important prognostic information (in particular the influence of tumour size and response to treatment on survival) as well as publishing two of the first papers that have actually gone to the trouble of computing tumour volume before and after treatment, though the figure in Sager's paper showing shrinkage of what is labelled as the uterus after chemotherapy does emphasise the limits of the technology at present.

I am sure that there can be little doubt that this book will be an important reference for the future, though its message to me is that we still have far to go if we are to improve on the figure of 30% 5-year survival for T3 bladder cancer which has been with us since the advent of megavoltage radiotherapy.

R.T.D. Oliver